# Effect of bariatric surgery on endometrial cancer regression as part of fertility sparing treatment

**DOI:** 10.1002/cnr2.1857

**Published:** 2023-07-05

**Authors:** Jinlin Lin, Weng Yan Ho, Qi Xuan Lim, Hui Xian Felicia Chin

**Affiliations:** ^1^ Consultant, Department of Surgery Changi General Hospital Singapore Singapore; ^2^ Consultant, Department of Gynaecological Oncology, Division of O&G KK Women's and Children Hospital Singapore Singapore; ^3^ Medical Officer, Department of Surgery Changi General Hospital Singapore Singapore

**Keywords:** bariatric surgery, endometrial cancer, fertility sparing

## Abstract

**Introduction:**

Obesity is a major risk factor in the development of endometrial cancer (EC) in young patients of reproductive age. Fertility sparing treatment is a viable option for a select group of patients with early EC, and involves systemic and intra‐uterine hormonal therapy. Weight loss has been associated with improved outcomes in this group. Bariatric surgery (BS) has been shown to be the most efficient and durable method of weight loss in obese patients. However, there is a paucity of data studying the benefit of BS as part of fertility sparing treatment.

**Methods:**

We present a retrospective case series of five patients who are undergoing fertility sparing treatment for early EC, who also underwent BS for treatment of obesity and related comorbidities. We aim to show early regression of EC for all the patients and also report on the other health benefits of BS.

**Results:**

All five patients in the series achieved regression of EC within 6 months of undergoing BS. They also achieved significant weight loss consistent with previous studies, and three patients who had comorbidities related to obesity had remission of these conditions. One of the patients with EC regression also managed to conceive with IVF (In‐vitro Fertilisation).

**Conclusion:**

Patients on fertility sparing treatment for early EC who underwent BS was associated with early regression within 6 months, significant weight loss and resolution of comorbidities. BS could be a promising component of fertility sparing treatment. Long term, prospective studies are required to confirm the benefits reported in this case series.

## 1 INTRODUCTION

Endometrial cancer (EC) is the second most common gynaecological cancer worldwide with 417 367 new cases diagnosed globally in 2020.^1^ Global estimates show rising incidence rates in both developed and developing countries.^2^ EC can be divided into 2 subtypes: type 1, the oestrogen‐dependent endometrioid type associated with obesity that accounts for up to 85% of ECs, and type 2, the non‐endometrioid subtypes that include serous, clear‐cell, undifferentiated carcinomas and malignant mixed Mullerian tumours and are typically not associated with obesity.^3^, ^4^ Although the majority of patients with EC are postmenopausal at diagnosis, about 20% of patients are diagnosed when they are still of reproductive age. The majority of these patients tend to present with low‐grade early stage tumours of the endometrioid subtype that are confined to the endometrium.^5^

The standard treatment for early EC is THBSO (Total Hysterectomy, Bilateral Salpingo‐Oopherectomy) with or without lymphadenectomy.^6^, ^7^ Following current standard surgical treatment, the 5‐year survival for EC are good, ranging from 74% to 91%, particularly for women diagnosed with low‐grade endometrioid tumours without lymph node involvement.^8^ However, given the current trends of women of reproductive age delaying childbearing^9^ and the rising incidence of EC amongst nulliparous women, an alternative treatment is necessary for patients who desire preservation of childbearing potential. Fertility sparing treatment for EC can be considered for a select group of patients who have FIGO (International Federation of Gynaecology and Obstetrics) grade 1 tumour of the endometrioid subtype, without myometrial invasion, lymph node involvement or distant metastasis. Even when all these criteria are met, patients should still be counselled that THBSO is the standard of care in early EC.^10^ This treatment approach mainly involves endocrine therapy with oral progestins, gonadotropin‐releasing hormone (GnRH) agonists or levonorgestrel‐releasing intra‐uterine devices. Patients on this treatment protocol require regular surveillance with endometrial biopsy until tumour regression.^11^ However, medical treatment alone for EC has the problems of long response time, unpredictable response and high recurrence rates.

Obesity is an established risk factor for EC, mainly due to the endogenous hyper‐estrogenic state it creates in a patient's body. The worldwide epidemic of obesity is likely to be a key factor in the increasing incidence of EC.^12^ Despite this clear link between obesity and EC, there is a paucity of data studying the effect of weight loss induced by bariatric surgery (BS) as part of the fertility sparing treatment. BS has been shown to be an effective treatment of obesity, producing sustained and significant weight loss, along with improvement in multiple obesity‐related co‐morbidities.^13^ At the tissue level, BS is associated with downregulation of pro‐proliferative signalling pathways, reduced endometrial growth, and spontaneous clearance of both latent and precursor endometrial neoplastic lesions.^14^ BS is also associated with reducing the odds of developing EC in obese women.^15^ Based on these factors, there is a strong biological rationale that weight loss induced from BS is an important factor that could contribute to successful regression of EC in patients on fertility sparing treatment. Additional benefits of BS for this group of patients include improvement in overall health from weight loss and improvement in fertility rates (from both natural conception and assisted reproduction) after fertility sparing treatment.^11^ In the event that these patients require surgical resection for EC in the future, weight loss also reduces peri‐operative risks and improves success rates for minimally invasive techniques.^16^

There is a paucity of studies that includes BS as a component of fertility sparing treatment of EC. The aim of this study is to provide a case series of patients on fertility sparing treatment who underwent BS for the treatment of morbid obesity. We aim to provide an observational analysis on the early regression of EC (within 6 months) with successful weight loss after and to report outcomes from BS including weight loss and improvement in related medical co‐morbidities.

## 2 METHODS

This is a single‐centre, retrospective case series. The patients included in this case series underwent BS for treatment of morbid obesity. In addition, they also had EC and were on fertility sparing treatment. Other inclusion criteria included (1) BMI (Body Mass Index) above 37.5 kg/m^2^, (2) BMI above 32.5 kg/m^2^, with co‐morbidities related to obesity and (3) patients of reproductive age who wish to preserve fertility. Exclusion criteria included (1) patients who did not undergo BS, (2) patients who underwent THBSO for treatment of EC and (3) patients on fertility sparing treatment, whose EC had regressed before BS. The study was approved by the Institutional Review Board (CIRB no: 2022/2195).

Starting from January 2021 to December 2022, from the hospital database, we identified five patients who fulfilled the inclusion criteria. After written consent was obtained from the patients, we collected data from electronic medical records (EMR). The data consisted of baseline patient characteristics, EC disease characteristics, fertility sparing treatment details, BS details and treatment outcome (in terms of EC regression, weight loss and improvement in metabolic conditions) after BS. The descriptive analysis of the data is show in the section below.

## 3 RESULTS

Five patients with confirmed EC were included in this case series. All of them were females at reproductive age who opted for fertility sparing treatment. Their age ranged between 27 and 37; the median patient age was 32. Three patients (60%) had co‐morbidities, which were related to obesity. The mean pre‐op weight was 109.54 kg and the mean pre‐op BMI was 40.73 kg/m^2^. Table 1 summarises the patient characteristics.

**TABLE 1:** Patient characteristics.

Patient	Age	Co‐morbidities	Pre‐op weight (kg)	Pre‐op BMI (kg/M^2^)
1	32	Asthma, DM	106.5	40.65
2	37	Hypertension, Hyperlipidemia, DM	113.1	40.10
3	27	None	89.8	38.80
4	35	Hyperlipidemia	106.4	40.00
5	29	None	131.9	44.10

Abbreviation: DM = Diabetes Mellitus.

Notably, there were two cases (Patients 4 and 5), where the endometrial cancer had previously regressed with hormonal treatment but subsequently recurred. In addition, Patient 1 had been treated with hormonal therapy for 12 months without regression. Patients 2 and 3 were diagnosed with endometrial cancer and started on hormonal treatment, but the tumour did not regress before they underwent BS, therefore they were included in the study. Four patients (80%) were diagnosed with hysteroscopy, dilatation and endometrial curettage (HDC), and one (20%) was diagnosed on endometrial sampling with the Explora Device. All patients had stage 1A, grade 1 (FIGO G1),^17^ endometroid carcinoma confined to endometrium. All patients had standard staging investigations after diagnosis with CT scan of the thorax and abdomen, as well as MRI scan of the pelvis. No evidence of myometrial invasion, lymph node or distant metastases was found after staging scans were performed for all patients. Patients 4 and 5 had repeat staging investigations before they were considered for fertility sparing treatment again, after EC recurrence. They had been offered THBSO after tumour recurrence but were still keen for fertility sparing treatment after counselling.

Before fertility sparing treatment was offered to all the patients, the cases were discussed in a multidisciplinary tumour board meeting, with concurrence from all treating specialists. All patients underwent hormonal therapy with oral Megestrol, gonadotropin‐releasing hormone agonists (Triptorelin and Leuprolide) as well as levonorgestrel‐releasing intra‐uterine device (Mirena), in accordance to the standard treatment pathway. After starting on treatment, patients underwent HDC on three monthly interval for surveillance. Table 2 summarises EC disease characteristics and hormonal therapy received.

**TABLE 2:** EC disease characteristics and hormonal therapy received.

Patient	Date of EC diagnosis	Method of diagnosis	Tumour grade	Tumour subtype	Systemic hormonal therapy	Intra‐uterine hormonal therapy	Duration of pre‐operative hormonal therapy
1	June 2020	HDC	G1	Endometroid	Megestrol, Triptorelin	Mirena	13 months
2	December 2020	HDC	G1	Endometroid	Megestrol, Triptorelin	Mirena	7 months
3	August 2021	Explora	G1	Endometroid	Megestrol, Triptorelin	Mirena	4 months
4	March 2016 (regressed July 2018, recurred October 2021)	HDC	G1	Endometroid	Megestrol, Triptorelin, **Leuprolide**	Mirena	6 months
5	March 2019 (regressed May 2020, recurred March 2022)	HDC	G1	Endometroid	Megestrol, Triptorelin, **Leuprolide**	Mirena	8 months

Abbreviation: HDC = Hysteroscopy, dilatation and endometrial curettage.

All the patients were referred to undergo BS as their BMI fulfilled criteria based on national healthcare guidelines. Pre‐operative preparation was done for all patients based on a standard pathway, including review by members of a multidisciplinary team, blood tests, upper gastro‐intestinal endoscopy and 2 weeks of meal replacement with a very low‐calorie diet (VLCD). All patients underwent laparoscopic sleeve gastrectomy (LSG) and there were no complications from BS. After BS, all patients had EC regression within 6 months based on histology on HDC (Table 3). After BS, the mean time taken for EC regression was 3.2 months. For three patients, 1, 3, 4, and there was normalisation of endometrial cells. The other two patients 2 and 5 had regression to complex hyperplasia. Patient 2 still had simple endometrial hyperplasia 10 months after BS, while follow‐up duration for patient 5 is still very short. Patients **were** instructed about contraception and to avoid conceiving for at least 12 months after BS. Two patients 1 and 3 underwent IVF (In‐vitro Fertilisation) 12 months after BS, with patient 1 successfully conceiving. There **were** no maternal or foetal complications during the gestation period up to the end of the follow‐up period.

**TABLE 3:** EC regression after BS.

Patient	Type of BS	Date of BS	Length of follow‐up (months)	Time to EC regression (months)	Histology at time at EC regression	Remarks
1	LSG	July 2021	19	1	Progesterone effect.	Maintained EC regression. Conceived via IVF 12 months after BS.
2	LSG	July 2021	19	2	Complex hyperplasia without atypia.	Regressed to simple hyperplasia 10 months after BS.
3	LSG	December 2021	14	6	Progesterone effect with endometritis.	Maintained EC regression. Started IVF 14 months after BS.
4	LSG	April 2022	10	5	Progesterone effect.	Maintained EC regression.
5	LSG	November 2022	3	2	Complex hyperplasia without atypia.	Maintained EC regression.

Abbreviations: BS = bariatric surgery; IVF = in‐vitro fertilisation; LSG = Laparoscopic Sleeve Gastrectomy.

After BS, all patients were followed‐up at three monthly intervals for the first 12 months, with regular blood tests to check for resolution of obesity‐related comorbidities and nutritional deficiency. All the patients were placed on vitamin, calcium and iron supplement as part of the standard treatment pathway. The weight loss and comorbidity resolution outcome are reported in Table 4. The mean total weight loss was 27.24 kg and mean percentage total weight loss was 25.28%. Patient 5 had lower weight loss compared to the rest of the patients due to the short follow‐up, and patients undergoing BS usually achieve maximal weight loss about 9 to 12 months post‐op. Besides the regression of endometrial cancer after BS, patients' 1, 2, 4, and comorbidities related to obesity went into remission, and they did not have to take medications to control these conditions anymore. Post‐remission, the patients did not need to continue with hormonal therapy.

**TABLE 4:** Weight loss and comorbidity resolution results after BS.

Patient	Type of BS	Date of BS	Pre‐op weight (kg)	Length of follow‐up (months)	Weight loss at end of follow‐up (kg)	Total body weight loss (%)	Resolution of comorbidities
1	LSG	July 2021	106.5	19	28.4	26.7	DM
2	LSG	July 2021	113.1	19	30.4	26.8	DM, Hypertension, Hyperlipidemia
3	LSG	December 2021	89.8	14	23.8	26.5	NA
4	LSG	April 2022	106.4	10	32.2	30.2	Hyperlipidemia
5	LSG	November 2022	131.9	3	21.4	16.2	N.A.

Abbreviations: BS = bariatric surgery; DM = diabetes Mellitus; LSG = Laparoscopic Sleeve Gastrectomy.

## 4 DISCUSSION

This study describes BS as a promising component in the fertility sparing treatment for patients who have early EC. There is a paucity of data for using BS as part of the fertility sparing treatment and this study adds to the body of knowledge regarding this subject. The only other study that discusses this subject is a prospective nonrandomized study conducted by Barr et. al^18^ who observed that weight loss improves the response rates in women with obesity and atypical hyperplasia or early EC undergoing fertility sparing treatment with intrauterine progestin. Patients who lost more than 10% of total body weight were nearly 4 times more likely to respond to intrauterine progestin than those who did not (OR 3.95 *p* = .02). In this study, BS was offered as a treatment for obese patients and resulted in a greater and more sustainable weight loss compared to nonsurgical treatment. No other studies have explored using BS as part of fertility sparing treatment of EC.

Morbid obesity is the underlying biological factor that drives the development of endometroid EC in young patients in the reproductive age group.^11^, ^12^ There is good evidence that obese patients who had BS have a reduced risk of developing endometrial cancer.^19^, ^20^, ^21^ Addressing this underlying factor with BS is a logical treatment strategy that can potentially improve the regression rates and reduce recurrence rates of EC. Indeed, we see two patients who had EC previously and had cancer regression with hormonal therapy. They had recurrence a few years after treatment. Another patient had a long treatment period with hormonal therapy, without regression of EC. This could possibly be because obesity, as the underlying driving factor for cellular proliferation and carcinogenesis,^13^ had not been addressed. Long term follow‐up and data is necessary to demonstrate if weight loss induced by BS results in reduced EC recurrence and survival benefit.

Fertility sparing treatment itself may exacerbate the problem of obesity. The most commonly reported side effects from hormonal therapy are appetite stimulation and weight gain. BS can help limit this by appetite suppression from reduced gastric volume and gut hormone alteration.

BS has been shown to be the most durable and effective treatment for obesity^13^ while improving the life expectancy and quality of life of obese patients.^22^, ^23^, ^24^ The improvement in physical and psychological health after BS provides benefit to this group of patients. Total weight loss is between 25% and 30%, which is consistent with other large‐scale studies. We also saw resolution of obesity related comorbidities, which could lead to improved health outcomes and reduced complications from cardiovascular diseases in the long term. There were no complications or adverse events in the patients in this study.

In addition, weight loss induced by BS improves the chances of fertility,^25^ both via natural conception or via ART (Assisted Reproductive Techniques). It also reduces maternal complications antenatally like pre‐eclampsia and gestational diabetes, and reduces risks in the peri‐partum period.^26^ We see that one of the patients had successfully conceived with IVF and had no maternal or foetal complications during the antenatal follow up period. Once the other patients pass the first 12 months after BS, where weight loss is rapid and extensive, they would be counselled to undergo ART to aid in conception.

The limitations of this study include the retrospective nature of the study design, the lack of a control group, the short follow‐up time and the small number of patients in the study group. The retrospective nature of the study design makes it prone to selection and measurement bias. The patients included in this study are only those that are treated in the centres in which the authors are based. In addition, the early cancer regression in this group of patient who chose to undergo BS may be due to other factors like higher compliance to the fertility sparing treatment or increased health seeking behaviour. Measurement bias can also result from incomplete or heterogeneous data from a lack of standard study protocol. This is partially mitigated by the fact that all the treatment received by the patient (both fertility sparing treatment for EC and BS) were according to a standard pathway, and all data collected were from the same comprehensive EMR system used in both public healthcare institutions. The outcomes measured were also objective in nature for example, histology proving that EC has regressed and weight loss measured in the outpatient clinic during follow‐up appointments. The lack of a control group prevents us from inferring a causal relationship between EC regression and BS. We are also unable to draw any conclusions about the longevity of the cancer regression due to the short follow‐up period.

## 5 CONCLUSION

In this retrospective case series, patients on fertility sparing treatment for early EC who underwent BS were associated with early cancer regression within 6 months. In addition, patients had significant weight loss and resolution of comorbidities. BS could be a promising component of fertility sparing treatment for early EC. The overall and metabolic health benefits from weight loss induced by BS in this study are consistent with current literature. Larger scale, prospective case controlled studies with longer follow‐up period is required to confirm the oncological benefit of BS for this purpose. This study could add to the body of evidence and raise awareness for this promising treatment strategy, while forming the background data for future prospective studies.

## AUTHOR CONTRIBUTIONS

**Jinlin Lin:** Conceptualization (equal); data curation (equal); formal analysis (equal); investigation (equal); methodology (equal); writing – original draft (equal); writing – review and editing (equal). **Weng Yan Ho:** Conceptualization (equal); methodology (equal); writing – review and editing (equal). **Qi Xuan Lim:** Writing – original draft (equal); writing – review and editing (equal). **Hui Xian Felicia Chin:** Conceptualization (equal); methodology (equal); writing – review and editing (equal).

## CONFLICT OF INTEREST STATEMENT

The authors report no conflict of interests in connection with this article.

### ETHICS STATEMENT

The study above has been approved by the Singhealth Institutional Review Board (CIRB no: 2022/2195), in accordance with the Human Biomedical Research Act 2015 of the Republic of Singapore. Explicit written consent was obtained from all subjects before inclusion in this study.

## Data Availability

Data available on request from the authors.
